# Investigating mitochondrial uncoupling protein 1 and leptin in the interplay of metabolic adaptation and inflammatory response of dairy cows during the peripartum period

**DOI:** 10.3389/fvets.2025.1628673

**Published:** 2025-09-01

**Authors:** Francesca Arfuso, Federica Arrigo, Maria Rizzo, Giulia Sisia, Enrico Fiore, Claudia Giannetto, Luigi Liotta, Giuseppe Piccione, Vincenzo Lopreiato

**Affiliations:** ^1^Department of Veterinary Sciences, University of Messina, Messina, Italy; ^2^Department of Animal Medicine, Productions and Health (MAPS), University of Padua, Legnaro, Italy

**Keywords:** inflammation, leptin, transition period, interleukins, UCP1, cows

## Abstract

**Introduction:**

This study investigated key metabolic markers and inflammatory responses in Simmental cows during the peripartum period, a physiologically demanding phase marked by metabolic and immune adjustments.

**Materials and methods:**

Ten Simmental cows were monitored, and blood samples were collected at various stages surrounding calving. Plasma concentrations of leptin, mitochondrial uncoupling protein 1 (UCP1), glucose, non-esterified fatty acids (NEFA), triglycerides, total cholesterol, β-OH-butyric acid (BHB), interleukin (IL)-1β, IL-6, and tumor necrosis factor α (TNFα) were measured.

**Results:**

Results indicated a decline in UCP1 from prepartum to calving, followed by a rise until three weeks postpartum. Leptin levels decreased prepartum and stabilized post-calving. IL-6 peaked at −7 days, while TNFα was elevated at −21 and −7 days compared to calving and subsequent days. Glucose levels were higher at −21 and −7 days, and NEFA increased from −21 until calving, then decreased. Total cholesterol was lower at calving and one day postpartum compared to earlier and later measurements. Triglycerides were elevated prepartum but decreased at calving. Leptin showed a positive correlation with TNFα, glucose, and triglycerides, and a negative correlation with NEFA. UCP1 values were negatively correlated with NEFA and positively correlated with total cholesterol.

**Discussion:**

These findings highlight the complex metabolic adjustments during the peripartum period in dairy cows. The observed fluctuations in UCP1 and leptin, along with their correlations with inflammatory and metabolic parameters, underline the intricate interplay between energy metabolism and immune function during this critical transition phase.

## 1 Introduction

The aptitude of an animal to yield a suitable reaction to a stimulus triggering a threat to homeostasis is crucial to survival. Homeostasis is known to be a fine coordinated control in metabolism of body tissues necessary to support a particular physiological state (1). It is well-accepted that stress condition suppresses immune competence of animal, increasing its susceptibility to diseases with negative consequence on both animal welfare and productive performance. Therefore, whether physiological or otherwise, any stressful factor involves the activation of different bodily systems which elicit a series of reactions interconnected with each other, with the aim of re-establishing the balance of the entire biological system to avoid the onset of a pathological state (2–4). The framework of this relationship is complex and it dynamically changes according to physiological condition. As such, the peripartum period of cows, also known as transition period, is characterized by metabolic and physiological adaptations in order to ensure successful pregnancy progress, parturition, and the onset of lactation (5–7). Under this scenario, a combination of multiple stressors markedly limits the ability of cows to achieve optimal performance (5–7) and increases the susceptibility to diseases and/or negatively impact the animal ability to overawed illness and recover. During the transition period, dairy cows experience major changes in energy metabolism (8). Adipose tissue is the first to act to maintain the body's energy balance as it is specialized in storing lipids and supplying fuel to the whole body whenever it is demanded (8). Besides a reserve organ, adipose tissue is also known to be an endocrine organ capable to produce hormones, peptides, and adipokines that affect both the energetic status and the immune system of animals (9). One of the most important hormones secreted by adipose tissue is leptin whose role in energetic homeostasis at central level has been largely described (10, 11). For a long time, leptin has been identified as the satiety hormone, while, nowadays, it is recognized as a signal of starvation, because a falling serum leptin concentration leads to neuro-humoral and behavioral changes, trying to preserve energy reserves for vital functions (12). In humans, higher levels of leptin may lead to a decrease in food intake and to an increase of energy expenditure by stimulating adipose tissue through the sympathetic nervous system and directly influencing the expression of the mitochondrial uncoupling protein 1 (UCP1) that lead to increasing lipolysis and fatty acid oxidation (13–16). Besides adipocytes, leptin is also produced by different tissues and organs, such as the stomach, skeletal muscle, pituitary cells and the placenta (17, 18). These various sites of leptin production explain the various functions that this hormone plays in different conditions. On this regards, the past 20 years of research on leptin have provided significant insights into the complex network that links metabolism, nutrition, reproduction as well as inflammation and immune functions (19–23). Noteworthy, it has been suggested that leptin could play a role in the modulator of immunological state of animals by acting on humoral and cellular immune responses (23). Leptin receptors belong to the family of class I cytokine receptors, and they have been found in neutrophils, monocytes, and lymphocytes (12, 24). Overall, studies carried out in humans and rodents suggest that leptin receptors were upregulated by pro-inflammatory signals (12). Leptin display a pro-inflammatory effect by the activation of pro-inflammatory cells, by the promotion of T-helper 1 responses, and by the mediation of the production of pro-inflammatory interleukins such as tumor necrosis factor-α (TNFα), interleukin (IL)-1, IL-2 and IL-6 (12, 23, 25). Consistent with this role of leptin in the mechanisms of immune response and host defense, circulating leptin levels are increased upon infectious and inflammatory stimuli (12). Noteworthy, the inflammatory stimuli could activate the browning process that leads to the beige adipocytes which increase the UCP1 expression augmenting non-shivering thermogenesis and metabolic capacity. As a matter of facts, the UCP1 plays important roles in metabolic and energy balance and regulation, in cold- and diet-induced thermogenesis and in decreasing oxidative stress associated with the pathogenesis of obesity (4). The browning process is spontaneously induced by tumor-secreted factors and IL-6 (26). Noteworthy, it has been suggested that UCP1 could influence inflammatory and pathogenic signaling in the liver (27). Although many research bodies dealt with several aspects of the cow's physiology during the peripartum period (5, 8, 28–36), there is a lack of information concerning the changes of leptin together with the UCP1, likely to be drivers of lipid metabolism, and with pro-inflammatory interleukins of peripartum cows. There are studies in the literature that have demonstrated the expression of UCP1 in adult cattle (37–41) though information on this protein in the bovine species during the peripartum period is scarce. In view of the above considerations and in order to provide insights on the field, the present study aimed to investigate both the changes in the serum concentration of the main markers of energetic balance (i.e., leptin, UCP1, glucose, NEFA, triglycerides, and total cholesterol), and, IL-1β, IL-6, and TNFα as markers of inflammation, in dairy cows during late pregnancy and the postpartum period. Furthermore, this study was also carried out to assess whether UCP1 and/or leptin values were correlated with the concentration of glucose, lipid metabolism indices and interleukins in periparturient cows in response to the physiological adaptation processes characterizing the transition from prepartum to postpartum period in cow.

## 2 Materials and methods

### 2.1 Animal management

The trial was carried out in accordance with ARRIVE guidelines and with Italian laws on animal experimentation (DL n. 26, 04/03/2014) and received an institutional approval from the Ethical Animal Care and Use Committee of the Magna Graecia University of Catanzaro (Protocol No. 302-5/5/2016). The farm owner was previously informed and the consent for animal use was obtained in compliance with the purposes and methods of the research.

A total of 10 multiparous Simmental dairy cows were enrolled in the study during the winter season, from a commercial dairy farm in Southern Italy (Calabria). Cows were monitored across the transition period (from −21 ± 4 to +21 days relative to parturition). Average temperature-humidity-index (42) during the trial was 53.4 (range: 41.0–65.7). The management of cows is the same described in our previous study (2). Cows were fed a total mixed ration (TMR) once daily at 07:00 a.m. both close up and lactation period (TMR was prepared separately for close-up and lactating cows); the diet composition used in close-up and early lactation periods is reported in Table 1. Cows were milked twice daily at 05:30 h and 17:30 h in a 8-A herringbone milking parlor. As described in previous studies (2, 43), the body condition scoring (BCS) was evaluated for each cow by the same operator using a 1–5 scale throughout the investigated period (21 ± 4 and 7 ± 3 days before expected calving, −21 and −7; within 4 h after calving, 0; at 1, 7, 15, and 21 days after calving, +1, +7, +15, +21).

**Table 1:** Ingredient and nutrient composition of close-up (from 21 days before parturition until calving) and early lactation (from parturition to 30 days of lactation) diets fed to Simmental dairy cows.

**Ingredient & nutrient**	**Close-up**	**Lactation**
DM, %	86.35	59.65
**Ingredient, % of DM**		
Alfalfa hay, second or later cuts	–	25.00
Grass hay	73.50	24.42
Corn grain, ground, dry	11.78	27.12
Commercial concentrate^a^	14.71	22.04
Minerals and vitamins	–	1.42
**Nutrient composition, % of DM**		
CP	11.16	15.05
Starch	11.12	23.09
Ether extract	2.22	2.80
Forage	75.02	52.23
NDF	49.08	36.87
ADF	25.40	21.94
ADL	3.74	4.29
NE_L_, Mcal/kg of DM^b^	1.46	1.63

^a^Contained: solvent-extracted flour of toasted soybean, solvent-extracted flour of sunflower, dried stillage corn, wheat bran, alfalfa flour, middling wheat, calcium carbonate, carob flour, fatty acid salts of palm oil, sodium chloride, molasses, phosphate dicalcium, sodium bicarbonate.

^b^According to NRC (87) and calculated using Razio-Best software of Università Cattolica del Sacro Cuore (Piacenza, Italy).

### 2.2 Blood sample collection and laboratory analysis

As previously described (2), blood samples were collected by jugular venipuncture into 9-ml lithium heparin vacutainer test tubes (Vacutest Kima srl, Arzergrande, PD, Italy) in the morning before TMR delivery. Thus, blood samples were collected at 21 ± 4 and 7 ± 3 days before expected calving (−21, −7), within 4 h after calving (0), and then at 1, 7, 15, and 21 days after calving (+1, +7, +15, +21). After blood collection, all samples were immediately cooled in an ice-water bath. The lithium heparin tubes were centrifuged at 1,900 × *g* for 16 min at 4 °C. Plasma was aliquoted and stored at −20 °C until analysis. The concentration of plasma concentration of leptin, UCP1, IL-1β, IL-6, and TNFα was assessed using ELISA kits specific for bovine species (Bovine Leptin Elisa kit, MyBioSource, Inc. San Diego, California, USA, Sensitivity 3.12 ng/ml; the intra- and the inter-assay coefficients of variation < 15%; Bovine UCP1 Elisa kit, MyBioSource, Inc. San Diego, California, USA, Sensitivity 0.05 ng/ml; the intra- and the inter-assay coefficients of variation were < 8 and < 12%; Bovine Interleukin 1 beta (IL-1β) Elisa kit, MyBioSource, Inc. San Diego, California, USA, Sensitivity 5 pg/ml; the intra- and the inter-assay coefficients of variation were < 8 and < 12%, respectively; Bovine Interleukin 6 (IL-6) Elisa kit, MyBioSource, Inc. San Diego, California, USA, Sensitivity 0.49 pg/ml; the intra- and the inter-assay coefficients of variation were < 10 and < 12%, respectively; Bovine Tumor Necrosis Factor α (TNFα) Elisa kit, MyBioSource, Inc. San Diego, California, USA, Sensitivity 20.8 pg/ml; the intra- and the inter-assay coefficients of variation were < 8 and < 12%, respectively) by means of a microtiter plate reader (Sirio, SEAC, Florence, Italy). According to previous study (2), all calibrators and samples were run in duplicate and samples exhibited parallel displacement to the standard curve for both ELISA analyses. The concentration of plasma triglycerides and total cholesterol was assessed using commercially available kits by means of an automated analyzer UV spectrophotometer (model Slim SEAC, Florence, Italy). Commercial kits were used to measure plasma glucose (Instrumentation Laboratory SpA, Milan, Italy) and non-esterified fatty acids (NEFA) levels (Wako, Chemicals GmbH, Neuss, Germany), whereas the concentration of β-OH-butyric acid (BHB) was measured with a kinetic UV method, based on the oxidation od D-3 hydroxybutyrate to acetoacetate by 3- Hydroxybutyrate dehydrogenase (kit Ranbut, RandoxLaboratories Limited, Crumlin, County Antrim, United Kingdom Randox, UK).

### 2.3 Statistical analysis

The data were analyzed with the software Prism v. 9.00 (GraphPad Software Ltd., San Diego, CA, USA, 2020). Cows served as their own controls and that animals were balanced for possible confounding variables (e.g., parity). Normal distribution of data was verified by the application of Shapiro–Wilk test. Data resulted normally distributed (*P* > 0.05) and they were subjected to one-way repeated measures analysis of variance (ANOVA). Bonferroni multiple comparison test was applied for *post-hoc* mean comparison. Pearson correlation analysis was applied to assess correlation among the concentration of leptin, UCP1, glucose, fats, BHB and inflammatory indices in cows throughout the experimental period. *P* values < 0.05 were considered statistically significant.

## 3 Results

**Figure 1:**
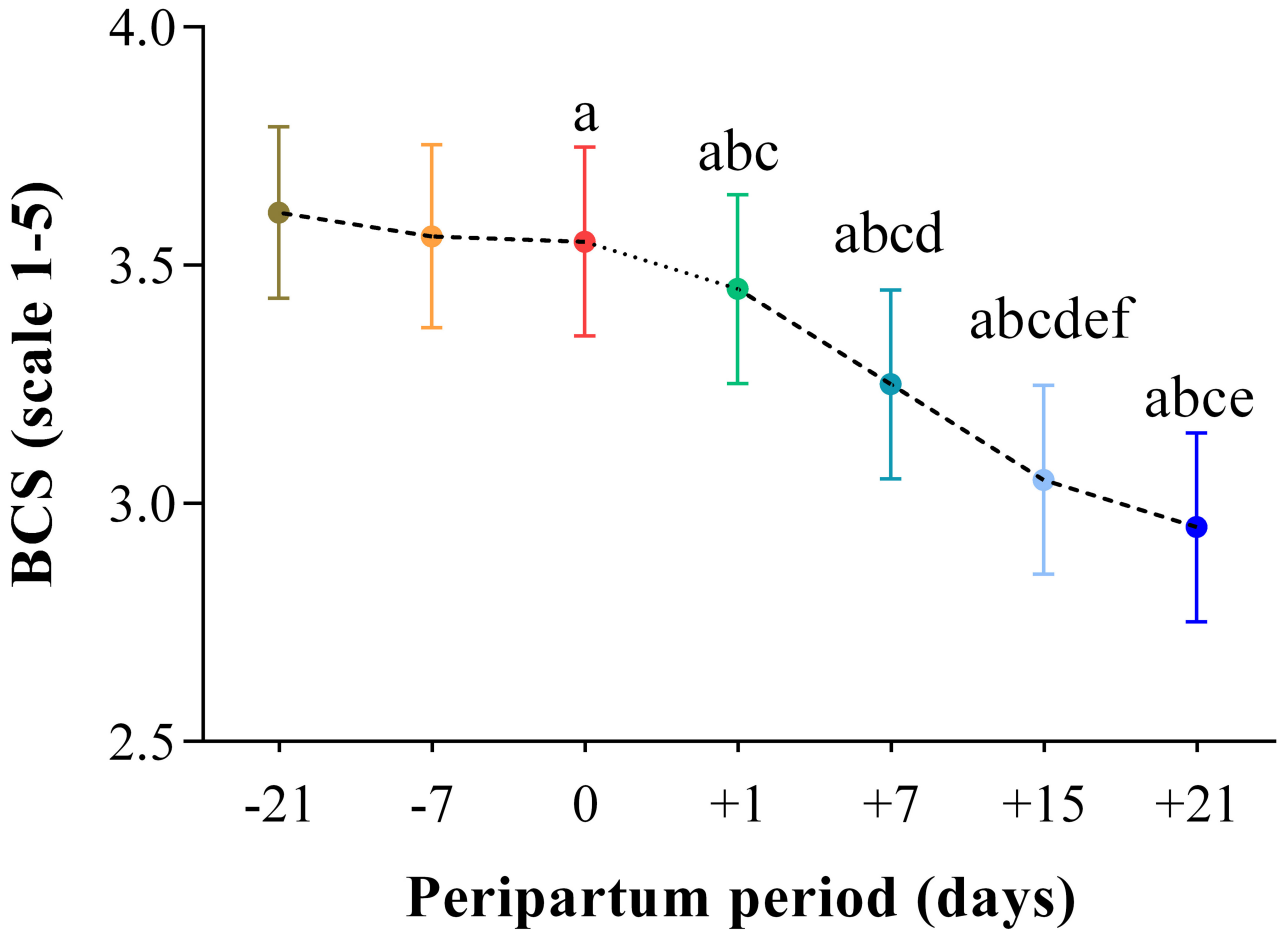
Mean values ± standard deviation (±SD) of body condition score (BCS) measured in cows during the investigated peripartum period (21 days before calving, −21; t days before calving, −7; calving day, 0; 1 day after calving, +1; 7 days after calving, +7; 15 days after calving, +15; 21 days after calving, +21) together with the relative statistical significances. Significances (effect of time; *P* < 0.05): a vs. −21; b vs. −7; c vs. 0; d vs. +1; e vs. +7; f vs. +21.

**Figure 2:**
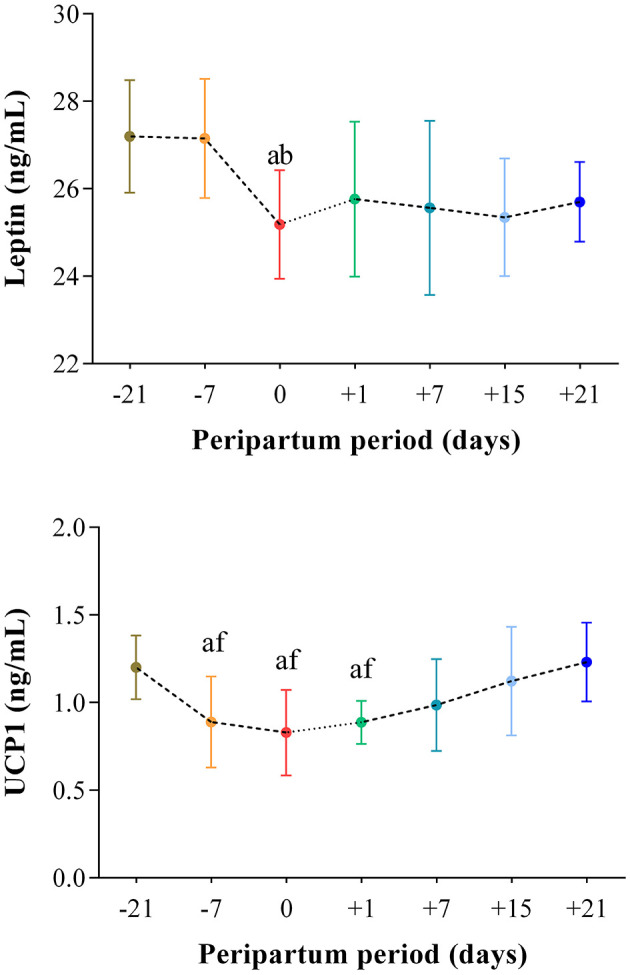
Mean values ± standard deviation (±SD) of plasma leptin and UCP1 measured in cows during the investigated peripartum period (21 days before calving, −21; t days before calving, −7; calving day, 0; 1 day after calving, +1; 7 days after calving, +7; 15 days after calving, +15; 21 days after calving, +21) together with the relative statistical significances. Significances (effect of time; *P* < 0.05): a vs. −21; b vs. −7; f vs. +21.

**Figure 3:**
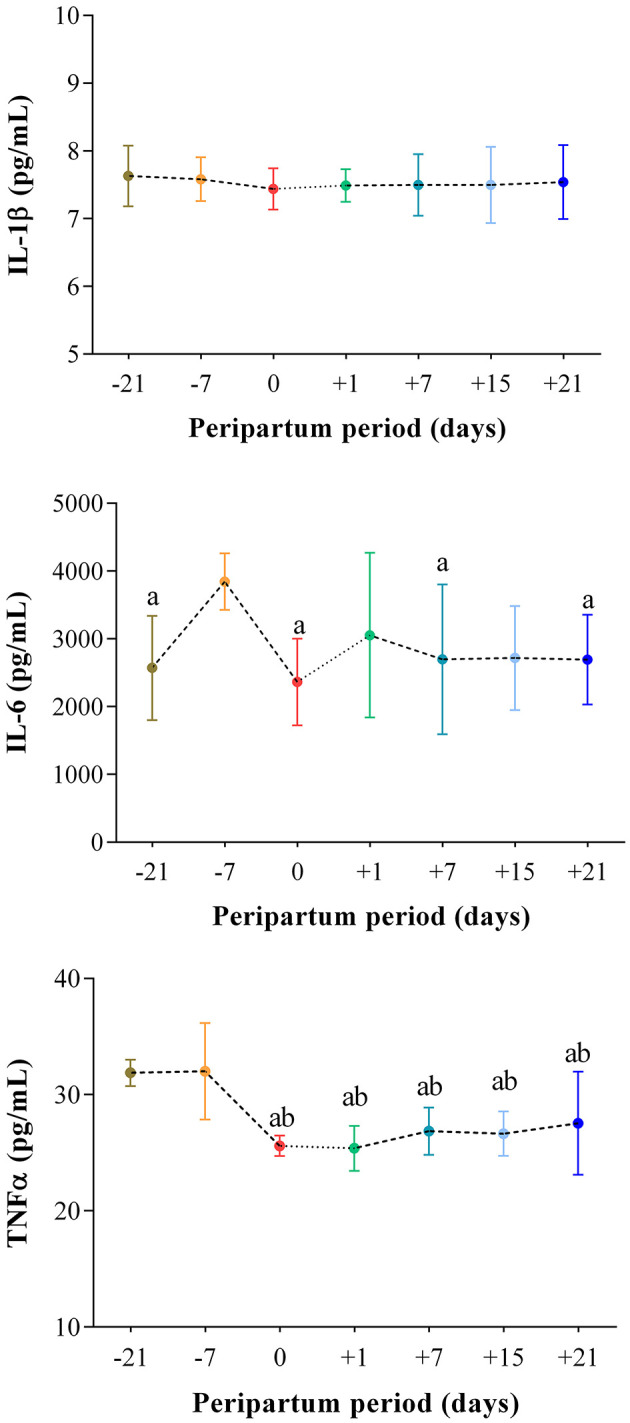
Mean values ± standard deviation (±SD) of plasma IL-1β, IL-6 and TNFα measured in cows during the investigated peripartum period (21 days before calving, −21; t days before calving, −7; calving day, 0; 1 day after calving, +1; 7 days after calving, +7; 15 days after calving, +15; 21 days after calving, +21) together with the relative statistical significances. Significances (effect of time; *P* < 0.05): a vs. −7; b vs. −21.

**Figure 4:**
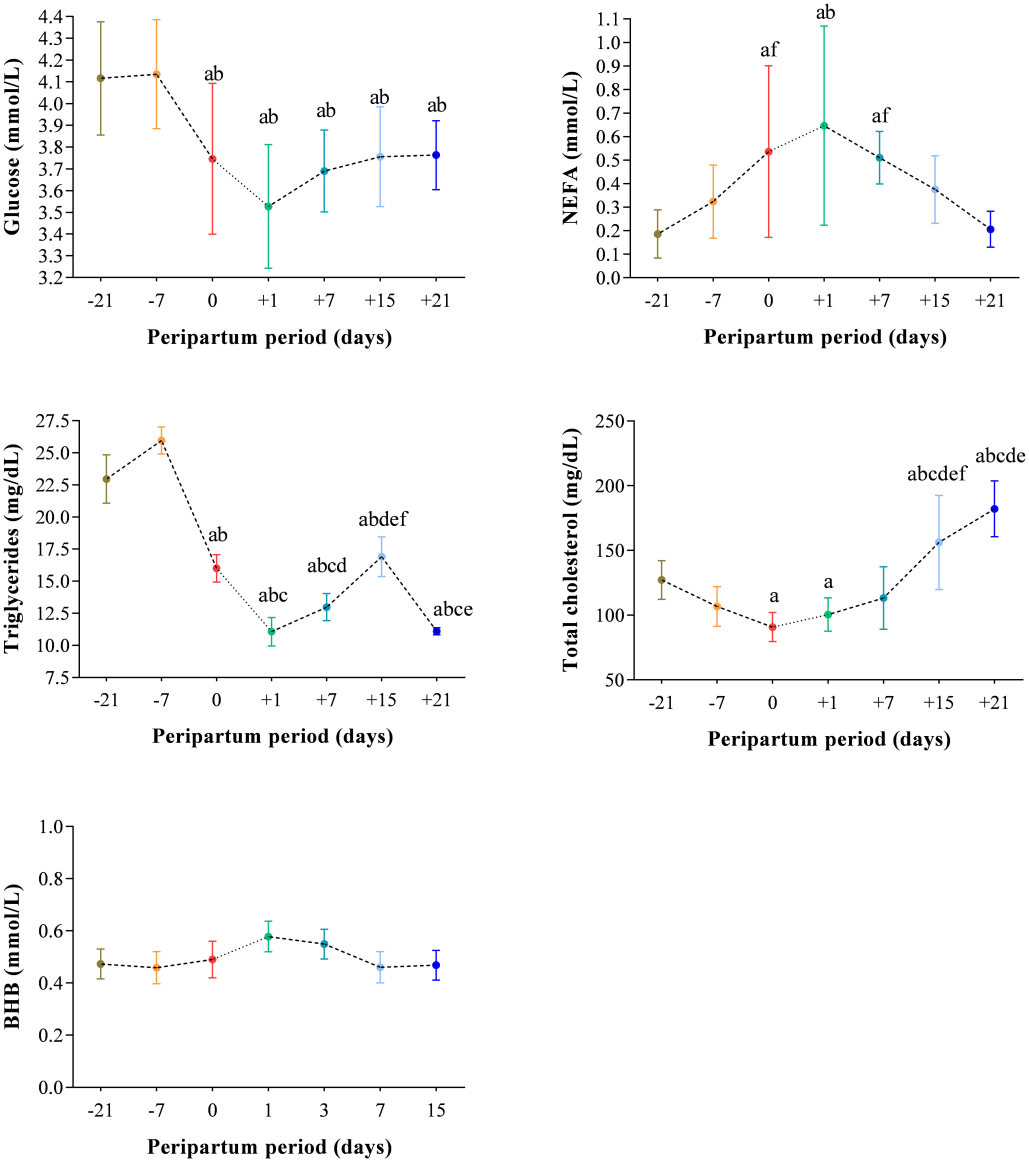
Mean values ± standard deviation (±SD) of plasma glucose, NEFA, triglycerides, total cholesterol and BHB measured in cows during the investigated peripartum period (21 days before calving, −21; t days before calving, −7; calving day, 0; 1 day after calving, +1; 7 days after calving, +7; 15 days after calving, +15; 21 days after calving, +21) together with the relative statistical significances. Significances (effect of time; *P* < 0.05): a vs. −21; b vs. −7; c vs. 0; d vs. +1; e vs. +7; f vs. +21.

The results obtained in the present study are expressed as mean values ± standard deviation (±SD). The BCS showed statistically significant changes over time (*P* < 0.05) with higher values at prepartum than postpartum time points (Figure 1). Except for IL-1β (*P* > 0.05) and BHB (*P* > 0.05), all the investigated parameters were affected by time relative to calving (*P* < 0.05). In particular, cows showed a decrease in UCP1 concentration from the prepartum period until calving followed by a rise until the third week after parturition (*P* < 0.05; Figure 2). Leptin values decreased from the prepartum period until calving (*P* < 0.05) and then remained constant throughout postpartum time (Figure 2). The plasma IL-6 concentration was higher at −7 compared with all the other peripartum time points (*P* < 0.05, Figure 3), whereas TNFα values were higher at prepartum time points (−21 and −7) than calving time (0) and postpartum period (+1, +7, and +21; *P* < 0.05, Figure 3). Throughout the monitored transition period, significant changes in the plasma concentration of glucose, NEFA, triglycerides and total cholesterol occurred. Specifically, glucose showed higher values at prepartum (−21 and −7) than calving (0) and postpartum period (+1, +7, and +21; *P* < 0.05, Figure 4). NEFA showed increasing trend from −21 until +1, thereafter the values decreased until +21 (*P* < 0.05; Figure 4). Triglyceride showed higher values at prepartum time than calving and the postpartum period (*P* < 0.05, Figure 4). As reported in Table 2, plasma leptin concentration showed a positive correlation with TNFα, glucose and triglycerides, and a negative correlation with NEFA (*P* < 0.05). The UCP1 values were negatively correlated with NEFA, and positively correlated with total cholesterol (*P* < 0.05).

**Table 2:** Coefficients of correlation among the plasma concentration of leptin and/or UCP1 and the plasma levels of glucose, NEFA, triglycerides, total cholesterol, TNFα, IL-1β, and IL-6 measured in cows during the investigated peripartum period (21 days before calving, −21; t days before calving, −7; calving day, 0; 1 day after calving, +1; 7 days after calving, +7; 15 days after calving, +15; 21 days after calving, +21).

	**Leptin (ng/ml)**			**UCP1 (ng/ml)**		
**Parameter analyzed**	**Pearson's** ***r***	***P*** **value**	***R*** **squared**	**Pearson's** ***r***	***P*** **value**	***R*** **squared**
Glucose (mmol/L)	**0.30**	**0.013**	**0.09**	0.03	0.834	0.00
NEFA (mmol/L)	**−0.32**	**0.007**	**0.10**	**−0.25**	**0.039**	**0.06**
Triglycerides (mg/dl)	**0.35**	**0.003**	**0.12**	**–**0.04	0.729	0.00
Total cholesterol (mg/dl)	**–**0.06	0.641	0.00	**0.47**	**0.000**	**0.22**
TNFα (ng/ml)	**0.34**	**0.004**	**0.11**	0.12	0.338	0.01
IL-1β (pg/ml)	−0.01	0.909	0.00	0.07	0.571	0.00
IL-6 (pg/ml)	0.10	0.414	0.01	−0.14	0.252	0.02
UCP1 (ng/ml)	0.13	0.266	0.02			

*P* values < 0.05 were considered statistically significant (in bold values).

## 4 Discussion

Though previous studies suggested a relationship among metabolic stress, immunosuppression, and inflammation in periparturient mammals, including cows (44, 45). To the best of authors knowledge, the current study for the first time evaluated whether leptin and UCP1 play a role in the interplay of lipid metabolism and inflammation in cows during the transition period. Overall, the results gathered in the present study confirm that transition period represents a challenging life phase for dairy cows whose energy balance undergoes major changes in response to the metabolic changes occurring during pregnancy and which allow the onset of lactation. In this study cows showed higher BCS values at the prepartum time followed by a decrease throughout postpartum time. This trend could be due to the changes in lipolysis and lipogenesis which are regulated to increase the lipid reserve during pregnancy, which will be utilized following parturition and the initiation of lactation (46–48). On this regard, the plasma levels of glucose showed a decreased trend in the postpartum time points likely due to the onset of lactation. A key factor in the first weeks after calving is the cow's ability to adequately respond to the continuous demand for glucose for milk production. It has been estimated that the demand of glucose after parturition is more than 500 g/day of the total splanchnic glucose supply predicted from digestible energy intake (46). The increase in blood levels of glucose can lead to a situation of insulin resistance in postpartum cows. This condition reduces the uptake of glucose by extra-mammary tissues that present insulin-dependent glucose transporters, in order to support milk production (47). The increase of plasma NEFA levels after calving contributes to this circumstance as well. According to the results gathered in this study, the NEFA showed increasing trend from −21 until +1, thereafter the values showed a decreasing trend up to 3 weeks after calving. Despite significant differences found in NEFA levels during the monitoring period, BHB levels did not show significant differences, which could indicate that the cows enrolled in the study have greater liver function that leads to better oxidation of NEFA. The blood concentration of NEFA is directly related to a situation of negative post-partum energy balance (48). The mechanism of lipolysis is controlled by endocrine signals mediated by epinephrine and norepinephrine, which increase in intensity during parturition and during the manifestation of any stressful factor. Consequently, the increase in NEFA around calving may be due to the stress of delivery (46). During early lactation, cows are in a negative energy balance and the body reserves are an important fuel supply to support the cows for milk synthesis (49, 50). This agrees with the changes in lipid profile of peripartum cows herein found. In particular, lower triglycerides values were found at calving and postpartum time points compared to prepartum ones. These changes agree with the results of previous studies (51, 52). The decrease in total cholesterol concentration throughout prepartum period might have resulted from the utilization of cholesterol by steroidogenic endocrine organs, including the ovaries and placenta for steroid hormone synthesis during pregnancy (53). It could be argued that UCP1 in mature animals is minimal/negligible as they have very little brown adipose tissue, which is the tissue directly associated with UCP1; however, it has been demonstrated that following a proper stimulation, white adipocytes can acquire typical features of brown fat cells including UCP1 expression (54) which may be used by organism to increase metabolic energy expenditure. This process is termed “browning” of white adipose tissue whose adipocytes, named as beige fat cells, acquire UCP1 expression ability and, thus thermogenic potential (54). On this regard, the presence of brown/beige adipocytes has been previously showed in the white fat depots of mature cattle (38). Noteworthy, the values of total cholesterol were positively correlated with the concentration of UCP1, suggesting a positive relationship between this protein and lipid metabolism in peripartum cows; whereas the NEFA resulted negatively correlated with UCP1. It is known that UCP1 dissipates the mitochondrial membrane potential, partially uncoupling substrate oxidation and oxidative phosphorylation and promoting the dissipation of cellular biochemical energy as heat (16). The main physiological activators of thermoregulation through UCP1 are the fatty acids resulting from hormone-stimulated lipolysis (16). This could explain the negative correlation found between this protein and NEFA in cows throughout the peripartum period. The results gathered in the current study showed a decreasing trend of UCP1 levels in transition cows from 3 weeks before expected parturition until calving and then, UCP1 increase up to 3 weeks later. This particular trend could be related to the actions of hormones working to ensure energy homeostasis during peripartum period. Although an explicable trend in plasma UCP1 levels in transition cows was observed in the present study, it is important to emphasize that the results herein gathered cannot claim that plasma UCP1 levels reflect adipose tissue activity without confirmatory data. As a matter of facts, further studies that analyze whether and how plasma UCP1 levels reflect the expression of this protein in adipose tissue in cows are recommended. The UCP1 are drivers of the thermogenic potential of adipose tissue and modulators of the distribution pattern of adipose tissue (55, 56). In mammals, the UCP1 expression is increased by estrogens (57) by an increase in sympathetic outflow on brown adipose tissue (58, 59). Noteworthy, the results obtained in this study seem to suggest that the mentioned effects of estrogens are abolished during pregnancy in cows. This speculation agrees with previous findings gathered from pregnant rats displaying a reduced thermogenesis and adipose tissue function (60). It could be hypothesized that pregnancy makes a state of resistance to the thermogenic action of estrogens, which likely contributes to gestational hyperphagia and adiposity to cope with the metabolic demands of embryonic development and the beginning of lactation (61). The plasma UCP1 levels increased in cows herein studied after calving, suggesting that, after parturition, estrogens recover their normal function of stimulating the thermogenic pathway (61). The increase in needs and speed of metabolic processes typical of postpartum results in a greater breakdown of estrogen and progesterone, thus modifying their feedback effect (62). The result of these processes can be observed in very productive cows with a high food intake, which have low levels of estrogen in circulation, therefore there will be less manifestation of estrous behavior (62, 63). One of the most important metabolic signals that regulate the relationship between nutrition and fertility is made up of leptin, a protein hormone synthesized in adipose tissue cells. The results obtained in the present study highlighted a decreasing trend of leptin concentration in transition cows from 3 weeks before expected parturition until calving and then it remains constant throughout the postpartum time. The blood level of leptin reflects the animal's energy status, in fact the loss of adipose tissue typical of postpartum reduces the production of this hormone (63). The plasma concentration of leptin presents major changes when the dietary plan undergoes sudden changes. Though fed did not change throughout the monitoring period and the feed intake was not measured due to its unreliability in the commercial farm setting, it is known to change drastically from the close-up period to early lactation, with the lowest intake typically occurring during the first 3 days after parturition.

It has been shown that in dairy cattle the greatest variations in leptin levels in the blood can be observed from 35 days before calving up to 56 days post-calving (64). The concentration of leptin is high at the beginning of the dry period and is reduced, even by 50%, in the peripartum period, and then remains at low levels even during the initial period of lactation. The reduction in plasma leptin concentration in dairy cows during the peripartum period can be attributed to two key factors, specifically, the onset of negative energy balance, positively correlated with a decline in levels of leptin, and the reduction of leptin gene expression in adipose tissue during early lactation (65). During the first weeks postpartum, insulin-dependent glucose uptake is partially compromised; glucose is considered to be the determining factor in the secretion of leptin by adipose tissue (66). Glucose exerts this effect by stimulating the production of metabolites (such as UDP-N-acetylglucosamine) which highlight the energy state of adipocytes and stimulate the secretion of leptin. This could justify the increasing trend of leptin after calving and also this would explain the positive correlation found between leptin and glucose. In cows at the beginning of lactation, the decreased blood levels of leptin reduce the response of peripheral tissues to the action of insulin, a key adaptive condition for promoting the use of blood glucose by the mammary gland (67). Cows in the peripartum period are characterized by a reduction in DMI mediated by the variation in the topography of the organs in the abdominal cavity and by metabolic changes. In particular, it seems that this variation in DMI is conditioned by the increase in circulating estrogen and continuous lipomobilization (68). It is known that the voluntary feed intake is reduced by the exogenous administration of leptin in small ruminants (59), thus, the low levels of leptin in the postpartum period should stimulate the continuous increase in ingestion. This would explain the positive correlation found between leptin and triglycerides and its negative correlation with NEFA suggesting a relationship among these parameters. As a matter of facts, it is established that the most important metabolic action of leptin consists in the regulation of adaptive response mechanisms to a state of undernutrition or in any case a lack of available food (69). Leptin also has a direct action on the functionality of the immune system (70). It seems that a leptin deficiency can cause an impairment of immunity mediated by T lymphocytes and in the regulation of macrophage activity (71). Moreover, an increase in leptin production during infection and inflammation has been previously demonstrated suggesting that this hormone is a part of the cytokine network which governs the inflammatory-immune response and defense mechanisms (12, 23–25). This hypothesis seems to be reinforced by the results gathered in this study showing a strong positive correlation between plasma leptin concentration and the pro-inflammatory cytokine TNFα measured in cows throughout the peripartum period. However, it must be taken into account that the correlation results herein found are indicative of possible associations, not necessarily causative pathways. TNFα is a potent pleiotropic pro-inflammatory cytokine playing a crucial role in inflammatory process and in enhancing neutrophil function such as neutrophil respiratory burst and lysosomal enzyme release in response to a wide range of soluble and particulate cell stimuli (72). According to the results obtained in the current study, which agree with previous findings in transition cows (73–75), high values of IL-6 and TNFα were observed during prepartum time points with a drop at calving and remaining lower up to 21 days after parturition. Though a high pro-inflammatory response in late pregnancy has been suggested as risky, in the current study no complications were detected in pregnant cows as previously observed in previous investigations (76). Moreover, the increased pro-inflammatory interleukin production starting from the third week before the expected calving could have a protective role by alerting and activating immune cells around the most stressful event of the transition period which is parturition (76). Indeed, the greater plasma concentrations of these interleukins could reflect a greater number and functionality of immune cells (2). On this regard, studies carried out in human and rodent clearly showed that during pregnancy the Th1 cell functions are restrained while the Th2 cell functions become dominant, activating maternal leukocytes which clearly secrete pro-inflammatory cytokines (77, 78). As previously hypothesized (2) the high pro-inflammatory interleukins during prepartum time could reflect a physiological trend during pregnancy and it could play a crucial role in preparing immune cells to promptly respond to critical moments such as parturition (2). Parturition is an inflammatory process in the utero-placental tissues and activation of the maternal immune response against the fetal membranes plays an important role in the separation of fetal membranes (74). The release of pro-inflammatory cytokines from uterine epithelial cells during parturition is important for the migration of inflammatory cells to the site of feto-maternal junction, which is a critical step for the fetal membrane separation (79). Pro-inflammatory cytokines including TNFα and IL-6 stimulate neutrophil and monocyte diapedesis, chemo attraction and promote phagocytosis (80). The TNFα and IL-6 concentration was remarkably reduced at calving and, although the reasons of this decline was not clear, many researches on this field attributed it to the strong increase of 17β-estradiol and cortisol likely to occur around calving (81–83). Despite its potential biological relevance, IL-1β levels did not vary in cows throughout the monitoring period; it should be considered that this lack of significance could be due to a high difference between data and/or timing of peak response as well as the peculiar secretion pathways of this interleukin. IL-1β is a potent pro-inflammatory cytokine produced by cells of the innate immune system. It is produced without a signal sequence and does not follow the conventional route of protein secretion, but rather employs one or more non-conventional pathways of secretion (84). It has been suggested that the secretion pathways part of one continuum of secretion, or a spectrum, where the routes of secretion employed are dictated by the strength of the inflammatory stimulus and, thus the levels of IL-1β required extracellularly to mount an effective inflammatory response (84). It has been suggested that bioactive IL-1b can be found in shed microvesicles from the plasma membrane (85), and, afterward, that it is also found in secreted exosomes (86). IL-1β has a very short half-life in plasma, therefore, it could be hypothesized that protected IL-1b is destined for sites distant to the inflammatory lesion (84).

## 5 Conclusions

The present study renews the knowledge currently available on the dynamic change of energetic balance together with inflammatory state of dairy cows during the peripartum period. The changes observed in plasma UCP1, leptin, glucose, and lipids levels of peripartum cows were the result of the hormonal and metabolic adaptations occurring during late pregnancy and lactation which is crucial to reward for energetic requirements of ovary, placenta, and mammary glands and, therefore, to protect cows against negative energy balance. Specifically, the lower plasma levels of UCP1 and leptin may play a role in counteracting the energy loss due to pregnancy requirements and milk production. This allows the cows to maintain a good BCS by decreasing energy expenditure and increasing lipolysis and fat utilization, as highlighted by the changes in plasma glucose and lipids concentration observed studied cows. Though the results obtained in the present study provide new insights to the current knowledge on the physiological response of cows during transition period, the limitations of the investigation (i.e., the lack of UCP1 tissue validation as well as the small sample size which may have influenced the detecting robust correlations) advocate the need for further studies enrolling a larger simple size and analyzing whether and how plasma UCP1 levels reflect the expression of this protein in adipose tissue in cows.

## Data Availability

The original contributions presented in the study are included in the article/supplementary material, further inquiries can be directed to the corresponding author.
